# Urban-rural-specific trend in prevalence of general and central obesity, and association with hypertension in Chinese adults, aged 18–65 years

**DOI:** 10.1186/s12889-019-7018-4

**Published:** 2019-05-30

**Authors:** Chi Shen, Zhongliang Zhou, Sha Lai, Xingxing Tao, Dantong Zhao, Wanyue Dong, Dan Li, Xin Lan, Jianmin Gao

**Affiliations:** 1 School of Public Policy and Administration, Xi’an Jiaotong University, No.28 Xianning West Road, Xi’an, 710049 Shaanxi China; 2 Health Science Center, Xi’an Jiaotong University, No.76 Yanta West Road, Xi’an, 710061 China

**Keywords:** Central obesity, General obesity, Hypertension, Urbanization

## Abstract

**Background:**

China has the largest obese population in the world, and the prevalence of central obesity is increasing dramatically in China. Moreover, the rapid economic growth of China in recent decades has led to rapid urbanization in rural China. However, studies comparing the prevalence trends of different types of obesity and the association of obesity with hypertension between urban and rural areas in China are very scarce, and most studies have focused only on the difference in the prevalence of overweight and general obesity or hypertension among rural and urban populations. Therefore, the focus of this study was to examine the shifts in the overall distribution of the prevalence of different types of obesity and to estimate the risk of hypertension in different types of obesity among urban and rural adults aged 18–65 years.

**Methods:**

Seven iterations of the China Health and Nutrition Survey (CHNS), conducted in 1993, 1997, 2000, 2004, 2006, 2009 and 2011, were used in this study. A total of 53,636 participants aged 18–65 years were included. Obesity was classified into three types based on body mass index (BMI) and waist circumference (WC). A log-binomial model was constructed to estimate the prevalence ratio (PR) of hypertension with three types of obesity.

**Results:**

The age-standardized prevalence of central obesity only, general obesity only, and both central and general obesity increased from 15.8, 0.2 and 2.9% in 1993 to 30.3, 0.9 and 10.3% in 2011, respectively. The prevalence of central obesity only (urban vs. rural: 20.8% vs. 13.4% in 1993, 29.6% vs. 30.6% in 2011) and both central and general obesity (urban vs. rural: 3.5% vs. 2.5% in 1993, 10.0% vs. 10.6% in 2011) in rural adults exceeded that in urban adults in 2011. Participants with both central and general obesity had the highest risk for incident hypertension compared with those with normal body measurements (adjusted PR, urban: 2.30 (95% *CI*, 2.01–2.63), rural: 2.50 (95% *CI*, 2.25–2.77)).

**Conclusions:**

Both WC and BMI should be considered measures of obesity and targeted in hypertension prevention. More attention should be paid to the incidence of central obesity in adults in rural China.

## Background

Obesity, a key cause of cardiovascular disease (CVD), is becoming a worldwide health concern [1]. According to the Chinese resident’s chronic disease and nutrition report in 2015, obesity rates among those 18 years old and above in China were 11.9% in 2012, which indicates that there were more than 124 million obese adults in China in 2012 [2]. Additionally, there were 292 million adults with hypertension in China in 2013–2014; in 2002, there were 153 million adults with hypertension [3]. However, in 2013 in China, the overall blood pressure control rates of adults with hypertension was only 9.7% [3]. Abnormally high blood pressure can lead serious health problem. In 2013, high systolic blood pressure caused 10.4 million deaths and 208.1 million disability-adjusted life years in the world [4].

Previous studies have revealed that obesity seemed to increase the risk of hypertension. In China, body mass index (BMI) is usually used to assess obesity [5]. However, obesity, as defined by BMI, is not a fitness indicator of body fat distribution [6]. Central obesity, measured by waist circumference (WC), has been shown to be a strong risk factor for the prevalence of hypertension and stroke [7]. Compared to individuals with a normal BMI, a population with central obesity has a higher risk of incident hypertension [8]. Additionally, with the improvement of living standards, young Chinese generations are facing more serious obesity problems, and younger cohorts have a higher prevalence of obesity than older cohorts [9, 10]. When these young people become older, those with central obesity will have a higher risk of developing hypertension later in life than those with general obesity [11].

However, studies comparing the trend in the prevalence of different types of obesity and the association with hypertension in urban and rural areas in China are scarce. Most studies have focused only on the difference in the prevalence of overweight and general obesity or hypertension among rural and urban populations [12, 13]. In recent decades, the economy of China has grown dramatically, leading to rapid urbanization in rural China. A previous study showed that China’s disease spectrum had significantly changed from communicable to noncommunicable diseases during China’s urbanization from 1995 to 2010 [14], and chronic health conditions, such as overweight and hypertension, were associated with urbanization [15]. Additional research is needed to explore the impact of urbanization in China on the prevalence of different types of obesity and the association with hypertension.

Therefore, this study focuses on examining the shifts in the overall distribution of the prevalence of different types of obesity and estimates the risk of hypertension with different types of obesity, while also comparing the discrepancy between urban and rural adults aged 18–65 years in China. We used cross-sectional data from a survey administered in China over 18 years to conduct our study.

## Methods

### Study design and population

We used data from the China Health and Nutrition Survey (CHNS) for our analysis. This survey is still ongoing and was completed in 1989, 1991, 1993, 1997, 2000, 2004, 2006, 2009, 2011 and 2015. The CHNS data and questionnaires can be used freely after registering at the official website (Data: https://www.cpc.unc.edu/projects/china/data, Questionnaires: https://www.cpc.unc.edu/projects/china/data/questionnaires). The main purpose of the survey was to investigate the health and nutrition status of Chinese individuals. The CHNS was conducted by the Carolina Population Center at the University of North Carolina at Chapel Hill and the National Institute of Nutrition and Food Safety at the Chinese Center for Disease Control and Prevention. A multistage, random cluster process was used to conduct the CHNS survey. The sample was taken in eight provinces (Liaoning, Shandong, Henan, Jiangsu, Hubei, Hunan, Guizhou, and Guangxi). In each province, counties and cities were stratified by income (low, middle and high), and four counties and two cities were randomly selected by a weighted sampling scheme. In the selected counties, villages and townships were randomly selected. In selected cities, urban and suburban neighborhoods were randomly selected. Twenty households were randomly selected, and all household members were interviewed in each community. Additional details of the CHNS can be found elsewhere [16]. The 1989 and 1991 surveys did not collect data on WC, and the 2015 survey only disclosed a part of the data, so this study examined data from 1993 to 2011. This study focused on adults aged 18–65 years. Participants who had missing data on WC, BMI, blood pressure (BP), age, urbanization, or gender or who were actively pregnant were excluded from the analysis. Our analysis included 53,636 participants (6782, 7288, 8227, 7243, 7236, 6668 and 10,192 participants in 1993, 1997, 2000, 2004, 2006, 2009 and 2011, respectively), with 18,216 from urban areas and 35,420 from rural areas.

### Study variables

Well-trained physicians conducted anthropometrical measurements, such as weight, height, WC, and BP, following a reference protocol recommended by the World Health Organization (WHO). The weight measurement was conducted with participants wearing lightweight clothing on a balance-beam scale that was accurate to 0.1 kg. The height measurement was conducted with participants barefoot on a portable stadiometer that was accurate to 0.1 cm [12]. The WC measurement was taken with a measuring tape placed at the midpoint between the lower margin of the arcus costalis on the midaxillary and the interiliac crest lines and was accurate to 0.1 cm [11]. Experienced physicians measured systolic and diastolic blood pressures three times on the right arm using a standard mercury sphygmomanometer. Before measurement, participants were required to have an initial five-minute, seated rest and a 30-s interval between cuff inflations; we used the mean value in this study [13]. Demographic and behavioral information, such as smoking, drinking, and urbanization, was collected by a structured questionnaire [16].

General obesity was defined as a BMI ≥ 28 kg/m^2^, which is recommended by the Working Group on Obesity in China [17]. Central obesity was defined as WC ≥ 90 cm for men and ≥ 80 cm for women, as recommended by the International Diabetes Federation [18]. Therefore, we classified obesity into three types: central obesity only (BMI < 28 kg/m^2^ and WC ≥ 90/80 cm), general obesity only (BMI ≥ 28 kg/m^2^ and WC < 90/80 cm) and both central and general obesity (BMI ≥ 28 kg/m^2^ and WC ≥ 90/80 cm). Participants with a BMI < 28 kg/m^2^ and a WC < 90/80 cm were defined as normal. Hypertension was defined as a self-reported doctor diagnosis of hypertension, a measured mean systolic blood pressure (SBP) ≥ 140 mmHg or diastolic blood pressure (DBP) ≥ 90 mmHg [19]. Participants who smoked cigarettes or drank alcohol during the previous year were recognized as smokers or drinkers, separately. In this study, the outcome variables were the prevalence of obesity and hypertension, and independent variables were smoking, drinking, gender, survey year, and age.

### Statistical analysis

In descriptive analyses, numeric variables were described as the means and standard deviation (SD), and character variables were presented as frequencies and percentages. All analyses were stratified by urbanization (urban vs. rural). The estimated prevalence was age-standardized to the 2010 Sixth National Population Census of Chinese adults by the direct method [20]. Student’s t-test was used to compare the difference in BMI, WC, SBP, DBP, and age between urban and rural individuals. A chi-square test was used to compare differences in the prevalence of hypertension between urban and rural areas. Cochran-Armitage trend testing was used to test the trends of the prevalence of three types of obese participants aged 18–65 years from 1993 to 2011. A multiple log-binomial mixed effect model was performed to estimate the prevalence ratio (PR) of hypertension with the three types of obesity. Models were used with adjustment for clustering effects (communities and households). Adjusted *PR* was estimated by adjusting smoking, drinking, gender, survey year, and age, which have been recognized as factors of hypertension [11, 21, 22]. All descriptive analyses, tests, and statistical models were conducted using SAS 9.4 (SAS, Cary, NC, USA), and visualization plots were conducted using the ‘ggplot2’ [23] and ‘ggridges’ [24] package in R software v.3.4.3.

## Results

### Sample characteristics and trends of BMI, WC, and BP distribution among populations in urban and rural areas from 1993 to 2011

**Fig. 1:**
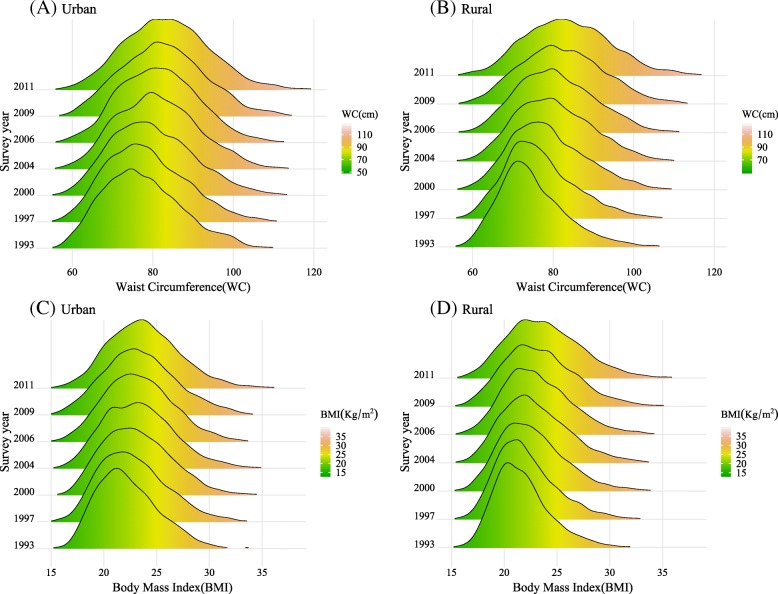
Shifts in the density curves of BMI and WC for Chinese adults aged 18–65 years, by urban and rural areas, 1993–2011

The characteristics of the participants are presented in Table 1. For all survey years, the number of female participants was slightly more than that of males. The mean age increased from 38.8 (SD = 12.87) years to 46.5 (SD = 11.99) years. For participants in both urban and rural areas, the anthropometric variables (BMI, WC, SBP, and DBP) increased notably over the past 18 years. We clearly found that the BMI and WC distribution curves among urban and rural participants shifted to the right from 1993 to 2011, as seen in Fig. 1. Before 2006, the BMI and WC of urban participants were higher than those of rural participants. The gap between urban and rural areas was statistically insignificant in 2009 and 2011.

**Table 1:** Characteristics of Chinese adults aged 18–65 years by survey year and urban and rural areas in 1993–2011

	Survey year						
	1993	1997	2000	2004	2006	2009	2011
All participants	6782	7288	8227	7243	7236	6668	10,192
Males n (%)	3235 (47.7)	3561 (48.9)	3936 (47.8)	3466 (47.9)	3418 (47.2)	3173 (47.6)	4777 (46.9)
Age (year)	38.8 (12.87)	39.8 (12.68)	41.3 (12.37)	43.7 (12.01)	44.7 (11.80)	45.6 (12.08)	46.5 (11.99)
BMI (kg/m2)	21.9 (2.81)	22.3 (3.04)	22.8 (3.16)	23.1 (3.28)	23.3 (3.50)	23.4 (3.40)	24 (4.18)
WC (cm)	75.6 (8.93)	77.3 (9.22)	79.1 (9.69)	80.5 (9.74)	81 (9.78)	82.4 (10.25)	83.7 (11.09)
SBP (mmHg)	113.9 (16.30)	116.8 (16.31)	117.7 (16.35)	119.9 (16.83)	119.5 (16.35)	121.9 (17.23)	122.5 (16.53)
DBP (mmHg)	75 (10.78)	76.5 (10.60)	77.1 (10.73)	78.3 (11.01)	78.5 (10.65)	79.8 (10.91)	79.2 (10.54)
Urban	2047	2457	2717	2435	2367	2108	4085
Males n (%)	972 (47.5)	1178 (47.9)	1291 (47.5)	1156 (47.5)	1121 (47.4)	995 (47.2)	1907 (46.7)
Age (year)	40 (13.10) ^**^	40.1 (12.74)	41.9 (12.46) ^*^	43.6 (12.36)	44.9 (12.12)	46 (12.25)	46.5 (12.22)
BMI (kg/m2)	22.3 (2.95) ^**^	22.8 (3.21) ^**^	23.1 (3.21) ^**^	23.4 (3.35) ^**^	23.4 (3.28) ^*^	23.5 (3.45)	24.1 (4.13)
WC (cm)	77.5 (9.80) ^**^	78.4 (9.82) ^**^	79.9 (10.15) ^*^	81.3 (10.01) ^*^	81.6 (9.73) ^**^	82.6 (10.26)	83.6 (10.94)
SBP (mmHg)	115.3 (17.26)	116.9 (16.91)	118 (16.42)	120.3 (16.95)	119.8 (15.99)	121.3 (16.68)	122.1 (15.58)
DBP (mmHg)	76.2 (11.36) ^*^	77 (10.76) ^**^	77.4 (10.96)	78.6 (10.68)	78.9 (10.27) ^*^	79.9 (10.50)	78.8 (9.54) ^**^
Rural	4735	4831	5510	4808	4869	4560	6107
Males n (%)	2263 (47.8)	2383 (49.3)	2645 (48.0)	2310 (48.0)	2297 (47.2)	2178 (47.8)	2870 (47.0)
Age (year)	38.3 (12.73)	39.7 (12.65)	41 (12.32)	43.7 (11.83)	44.7 (11.65)	45.5 (11.99)	46.4 (11.83)
BMI (kg/m2)	21.7 (2.73)	22.1 (2.92)	22.7 (3.13)	23 (3.23)	23.2 (3.59)	23.3 (3.37)	24 (4.22)
WC (cm)	74.8 (8.39)	76.7 (8.85)	78.8 (9.43)	80.2 (9.58)	80.7 (9.79)	82.2 (10.25)	83.7 (11.19)
SBP (mmHg)	113.2 (15.82)	116.7 (15.99)	117.5 (16.31)	119.6 (16.76)	119.4 (16.53)	122.2 (17.47)	122.7 (17.13)
DBP (mmHg)	74.5 (10.47)	76.2 (10.52)	76.9 (10.60)	78.2 (11.17)	78.2 (10.82)	79.7 (11.10)	79.5 (11.16)

Values presented as numbers for arbitrary values and as the mean (SD) or n (%) for other variables

Student’s t-test was applied to test the difference between urban and rural areas, ^*^ significant at 0.05, ^**^ significant at 0.01

### Prevalence trends of three types of obesity among urban and rural populations from 1993 to 2011

In Table 2, we presented the age-standardized prevalence of central obesity only, general obesity only, and both central and general obesity by survey year. The age-standardized prevalence of central obesity only, general obesity only, and both central and general obesity increased from 15.8, 0.2 and 2.9% in 1993 to 30.3, 0.9 and 10.3% in 2011, respectively. The highest prevalence was observed in participants with central obesity only (urban: 29.6%, rural: 30.6%, in 2011). The prevalence of participants with general obesity only (urban: 1.1%, rural: 0.8%, in 2011) was the lowest. Meanwhile, the gap between the prevalence of central only obesity and both central and general obesity between urban and rural participants narrowed from 1993 to 2011. Before 2006, the participants in urban areas had a higher prevalence of central obesity only than that in participants in rural areas, but the prevalence in rural participants increased in 2009 and 2011. However, the prevalence of general obesity was still higher in urban participants than that in rural participants.

**Table 2:** Age-standardized prevalence (95% *CI*) of three types of obesity in Chinese adults, aged 18–65 years, by survey year and urban and rural areas in 1993–2011

	Survey year							*P* for trend ^a^
	1993	1997	2000	2004	2006	2009	2011	
All participants								
Central obesity only	15.8 (15.5–16.0)	18.0 (17.7–18.2)	21.8 (21.6–22.1)	23.7 (23.5–24.0)	25.0 (24.8–25.3)	27.9 (27.6–28.2)	30.3 (30.0–30.6)	<.0001
General obesity only	0.2 (0.2–0.3)	0.3 (0.2–0.3)	0.4 (0.4–0.4)	0.4 (0.4–0.5)	0.6 (0.5–0.6)	0.4 (0.4–0.5)	0.9 (0.9–1.0)	<.0001
Both central and general obesity	2.9 (2.8–3.0)	4.4 (4.3–4.6)	5.6 (5.5–5.8)	6.4 (6.3–6.6)	6.4 (6.2–6.5)	8.1 (8.0–8.3)	10.3 (10.1–10.5)	<.0001
Urban								
Central obesity only	20.8 (20.6–21.1) ^**^	21.3 (21.0–21.6) ^**^	23.2 (23–23.5) ^**^	24.9 (24.6–25.2) ^**^	26.2 (25.9–26.4) ^**^	27.5 (27.2–27.8) ^*^	29.6 (29.3–29.9) ^**^	<.0001
General obesity only	0.1 (0.1–0.2) ^**^	0.4 (0.3–0.4) ^**^	0.5 (0.5–0.6) ^**^	0.3 (0.3–0.3) ^**^	0.7 (0.6–0.7) ^**^	0.9 (0.8–0.9) ^**^	1.1 (1.1–1.2) ^**^	<.0001
Both central and general obesity	3.5 (3.4–3.6) ^**^	6.1 (5.9–6.2) ^**^	6.6 (6.4–6.7) ^**^	7.5 (7.3–7.6) ^**^	6.5 (6.4–6.7) ^*^	8.7 (8.6–8.9) ^**^	10.0 (9.8–10.2) ^**^	<.0001
Rural								
Central obesity only	13.4 (13.2–13.6)	16.3 (16.0–16.5)	21.1 (20.8–21.3)	23.2 (22.9–23.4)	24.5 (24.2–24.7)	28.0 (27.7–28.3)	30.6 (30.3–30.9)	<.0001
General obesity only	0.3 (0.2–0.3)	0.2 (0.2–0.2)	0.3 (0.3–0.4)	0.5 (0.5–0.5)	0.5 (0.4–0.5)	0.2 (0.2–0.3)	0.8 (0.7–0.9)	<.0001
Both central and general obesity	2.5 (2.4–2.6)	3.6 (3.5–3.7)	5.2 (5.0–5.3)	5.8 (5.7–6.0)	6.3 (6.1–6.4)	7.9 (7.7–8.0)	10.6 (10.4–10.8)	<.0001

*CI* confidence interval

A chi-square test was applied to test the difference between urban and rural, ^*^ significant at 0.05, ^**^ significant at 0.01

^a^Trends in the prevalence of three types of obesity from 1993 to 2011 were assessed by Cochran-Armitage trend testing

### Prevalence ratio of hypertension with different types of obesity among participants in urban and rural areas

Table 3 presents the prevalence ratio (PR) and adjusted PR of hypertension with different types of obesity among the populations in urban and rural areas. For both urban and rural participants, both central and general obesity had higher risk for incident hypertension than did normal controls (urban: PR = 3.46, 95% *CI*, 3.16–3.79; rural: PR = 3.64, 95% *CI*, 3.40–3.90). After adjustment for gender, smoking status, drinking status, age, and survey year (urban: adjusted PR = 2.30, 95% *CI*, 2.01–2.63; rural: adjusted PR = 2.50, 95% *CI*, 2.25–2.77), compared with normal subjects, urban participants with central obesity (adjusted PR = 1.82, 95% *CI*, 1.62–2.04) had a lower risk for incident hypertension than did those with general obesity (adjusted PR = 2.13, 95% *CI*, 1.48–3.05). However, compared with normal subjects, rural participants with central obesity (adjusted PR = 1.78, 95% *CI*, 1.62–1.94) had a higher risk for incident hypertension than did those with general obesity (adjusted PR = 1.50, 95% *CI*, 0.96–2.33).

**Table 3:** Prevalence ratios of hypertension with different types of obesity in urban and rural areas according to the log-binomial mixed effects model

	Urban		Rural	
	PR(95% *CI*)	Adjusted PR(95% *CI*)	PR(95% *CI*)	Adjusted PR(95% *CI*)
Normal (Ref)	1	1	1	1
Central obesity only	2.22 (2.06–2.38)^**^	1.82 (1.62–2.04) ^**^	2.03 (1.92–2.15) ^**^	1.78 (1.62–1.94) ^**^
General obesity only	2.56 (1.94–3.39) ^**^	2.13 (1.48–3.05) ^**^	1.79 (1.31–2.45) ^**^	1.50 (0.96–2.33)
Both central and general obesity	3.46 (3.16–3.79) ^**^	2.30 (2.01–2.63) ^**^	3.64 (3.40–3.90) ^**^	2.50 (2.25–2.77) ^**^

*CI* confidence interval, ^*^ significant at 0.05, ^**^ significant at 0.01

^a^Normal indicates participants with a BMI < 28 kg/m^2^ and WC < 90/80 cm

^b^Adjusted for smoking, drinking, gender, survey year, age and clustering effects (communities and households)

## Discussion

In our study, we demonstrated that the age-standardized prevalence of central obesity only, general obesity only, and both central and general obesity all increased significantly in Chinese adults from 1993 to 2011. The prevalence of participants with central obesity only was the highest for the three types of obesity. Additionally, the prevalence of central obesity only and both central and general obesity in adults in rural areas exceeded that of adults in urban areas in 2011. Participants with both central and general obesity had the highest risk of incident hypertension compared with those with normal BMIs and WCs.

This study showed that an upward trend was noted in the distribution of BMIs and WCs in Chinese adults aged 18–65 years. The mean BMI increased from 21.9 kg/m^2^ to 24.0 kg/m^2^, and the mean WC increased from 75.6 cm to 83.7 cm in 1993 and 2011, respectively. These results are in line with previous studies [9–11]. Distribution curves of BMIs and WCs shifted to the right, which indicated that the population suffered a higher proportion of obesity and overweight. As we found, the prevalence of obesity, whether defined by BMI or WC, has increased significantly over the past 18 years in China, particularly central obesity. The rising trend is similar in Korean men, whose prevalence rate of abdominal obesity increased from 22.1% in 1998 to 27.5% in 2007 [25]. In addition, in the three types of obesity, we revealed that the prevalence of central obesity with a normal or abnormal BMI (30.3 and 10.3% in 2011) was far higher than that of general obesity with a normal WC (0.9% in 2011), and no difference was found between participants from urban and rural areas. This constitution of different types of obesity has also been reported in a previous study, in which the constituent ratio of subjects with an exclusive BMI ≥ 28 kg/m^2^ was only 0.9% in 2009 [8]. This finding suggests that it may be more accurate to combine WC and BMI when screening for obesity.

One of the concerns in our study was whether there was a similarity or difference in the prevalence of obesity among adults in urban and rural areas in China. Notably, the prevalence of central obesity increased more quickly among adults in rural areas than among adults in urban areas in this study. This result seems to be consistent with previous studies that found that the prevalence of central obesity of residents in rural areas increased more rapidly than that of residents in urban areas [8, 26]. However, we have some new findings. First, the gap between the prevalence of central obesity only and both central and general obesity between adults in urban and rural areas narrowed from 1993 to 2011. Second, the overall prevalence of central obesity in adults in rural areas was beyond that in adults in urban areas in 2011. These findings may be attributed to the changes in China’s social and economic structure led by China’s urbanization [27]. A previous observation confirmed that chronic health conditions, such as overweight, are associated with modernization and affluence, and the appearance of these conditions is no longer restricted to urban populations [15]. In China, the average food consumption per person in rural households increased from 890.3 Yuan in 1997 to 2323.9 Yuan in 2012, and the Engel coefficients of urban and rural households were 46.6 and 55.1% in 1997 and 36.2 and 39.3% in 2012, respectively [28]. Along with urbanization, the food consumption capacity of adults in rural areas has developed rapidly. Therefore, it is possible that accelerated health decline, such as the prevalence of central obesity in more urbanized areas, is exacerbated by a high-fat diet and decreased physical activity [29].

Another aim of our study was to estimate the risk of hypertension with the three types of obesity and to compare the differences between adults in urban and rural areas. The positive relationship between hypertension and obesity has been reported widely in many studies [11, 30–33]. In Chinese adults, BMI is highly associated with high blood pressure [32], and changes in BMI show a dose-response relationship with incident hypertension [33]. WC provides a unique indicator of body fat distribution and has often been used as a reasonable predictor of the risk of hypertension [34]. Previous studies have reported that central obesity also raises the risk of incident hypertension in later life [11] and leads to a higher risk of incident hypertension compared with that of individuals with a normal BMI [8]. However, studies have not investigated whether a population with central obesity has a higher risk of incident hypertension compared with a population with general obesity. Limited information from previous studies is available [8]; however, our results expanded on this information. We found that the risk of hypertension among the three types of obesity in adults in urban areas is as follows: both central and general obesity > general obesity only > central obesity only and in adults in rural areas is both central and general obesity > central obesity only > general obesity only. This indicates that central obesity not only is an independent risk of hypertension but also increases the risk when accompanied by general obesity.

Several limitations exist in our study. First, as a limitation of the data, we cannot provide exact explanations for the prevalence of central obesity among adults in rural areas exceeding that of adults in urban areas in 2011. Second, the risk of hypertension with central obesity only and general obesity only is not consistent in adults in urban and rural areas. If central obesity is commonly used as a reasonable predictor of the risk of incident hypertension in Chinese adults, which should be consistent in urban and rural adults, future research may be needed to examine the reasons for this. Third, although our sample size was large and the participants were from eight provinces in China, the sample was still only a part of China, and a broader national study should be conducted to support the results. Fourth, the data of CHNS used in this study is cross-sectional data, the causal inference is weak, and it is difficult to deeply explore the relationship between obesity and hypertension. Fifth, the most recent data in our study was from 2011, but China’s urban and rural areas are still very much changing, and an updated study should continue to pay attention to and observe the difference in trends of residents of urban and rural areas in China.

## Conclusion

This study has shown that the prevalence of central obesity increased notably in Chinese adults aged 18–65 years with normal or abnormal BMI from 1993 to 2011. In addition, the prevalence of central obesity increased more rapidly in adults in rural areas than it did in adults in urban areas. The prevalence central obesity only and both central and general obesity in adults in rural areas exceeded that in adults in urban areas in 2011. Subjects with both central and general obesity had the highest risk of incident hypertension despite normal BMI or WC. Therefore, both WC and BMI should be considered measures of obesity and targeted in hypertension prevention. More attention should be paid to the incidence of central obesity in adults in rural areas.

## Data Availability

The datasets used in this study are available from the China Health and Nutrition Survey (http://www.cpc.unc.edu/projects/china/data/datasets/data_downloads/longitudinal.). The CHNS data and questionnaires are publically available, everyone can use freely after registering at the official website.

## Abbreviations

BMI: Body Mass Index
BP: Blood Pressure
CHNS: China Health and Nutrition Survey
CI: Confidence Interval
CVD: Cardiovascular Disease
DBP: Diastolic Blood Pressure
PR: Prevalence Ratio
SBP: Systolic Blood Pressure
SD: Standard Deviation
WC: Waist Circumference
WHO: World Health Organization
